# Allometric scaling of metabolic rate and cardiorespiratory variables in aquatic and terrestrial mammals

**DOI:** 10.14814/phy2.15698

**Published:** 2023-06-04

**Authors:** Rebecca S. He, Stacy De Ruiter, Tristan Westover, Jason A. Somarelli, Ashley M. Blawas, Divya L. Dayanidhi, Ana Singh, Benjamin Steves, Samantha Driesinga, Lewis G. Halsey, Andreas Fahlman

**Affiliations:** ^1^ Duke University Marine Laboratory Nicholas School of the Environment Beaufort North Carolina USA; ^2^ Department of Biology Duke University Durham North Carolina USA; ^3^ Department of Mathematics and Statistics Calvin University Grand Rapids Michigan USA; ^4^ Department of Medicine Duke University Medical Center Durham North Carolina USA; ^5^ School of Life and Health Sciences University of Roehampton London UK; ^6^ Fundación Oceanogràfic de la Comunitat Valenciana Valencia Spain; ^7^ Kolmarden Wildlife Park Kolmarden Sweden; ^8^ Linkoping University, IFM Linköping Sweden

**Keywords:** basal metabolic rate, body mass, breathing frequency, heart rate, stroke volume, tidal volume

## Abstract

While basal metabolic rate (BMR) scales proportionally with body mass (*M*_b_), it remains unclear whether the relationship differs between mammals from aquatic and terrestrial habitats. We hypothesized that differences in BMR allometry would be reflected in similar differences in scaling of O_2_ delivery pathways through the cardiorespiratory system. We performed a comparative analysis of BMR across 63 mammalian species (20 aquatic, 43 terrestrial) with a *M*_b_ range from 10 kg to 5318 kg. Our results revealed elevated BMRs in small (>10 kg and <100 kg) aquatic mammals compared to small terrestrial mammals. The results demonstrated that minute ventilation, that is, tidal volume (*V*_T_)·breathing frequency (*f*_R_), as well as cardiac output, that is, stroke volume·heart rate, do not differ between the two habitats. We found that the “aquatic breathing strategy”, characterized by higher *V*_T_ and lower *f*_R_ resulting in a more effective gas exchange, and by elevated blood hemoglobin concentrations resulting in a higher volume of O_2_ for the same volume of blood, supported elevated metabolic requirements in aquatic mammals. The results from this study provide a possible explanation of how differences in gas exchange may serve energy demands in aquatic versus terrestrial mammals.

## 1 INTRODUCTION

Basal metabolic rate (BMR) is a fundamental metric in comparative physiology that describes the baseline energetic requirements of organisms and is useful for understanding how changes in an organism's environment affect its energy expenditure (Kleiber, 1961). It is well‐known that BMR scales with body mass (*M*_b_) as BMR *= cM*_b_^*n*^, where *n* is the allometric mass‐exponent and *c* a constant. However, there is still considerable controversy over the value of the allometric mass‐exponent (Benedict & Ritzman, 1935; Darveau et al., 2002; Kleiber, 1947; Rubner, 1865; West et al., 2002; White, 2010), where values between 0.67 and 0.75 have been the most commonly cited, and a number of theoretical models have been developed that support these estimates (Darveau et al., 2002; West et al., 1997; West et al., 2002; White & Seymour, 2003). There are also studies that argue there is no single universal mass‐exponent and that different groups of species follow different scaling relationships (Darveau et al., 2002; Kolokotrones et al., 2010; White, 2010), perhaps, for example, due to different diets or ambient temperatures—as seen in the binturong (*Arctictis binturong*) and the red panda (*Ailurus fulgens*) (Upham et al., 2019, McNab, 2008, McNab, 1988, Naya et al., 2018).

A number of studies have measured BMR in aquatic mammals and have proposed that semi‐ and fully aquatic species have higher metabolic requirements than terrestrial mammals (Hart et al., 1959; Irving et al., 1935; Irving et al., 1941; Irving & Hart, 1957; Kanwisher & Ridgway, 1983; Kanwisher & Sundness, 1965; Scholander, 1940; Scholander et al., 1942; Scholander et al., 1950; Sergeant, 1973; Snyder, 1983; South et al., 1976; Williams et al., 2001). However, other studies oppose this view, presenting data that suggest the BMR of aquatic mammals is not elevated (Gallivan & Ronald, 1979; Lavigne et al., 1986; Pedersen et al., 2020; Rosen & Trites, 2013; Schmitz & Lavigne, 1984; Worthy et al., 2013). There are a number of potential reasons for this discrepancy. First, BMR is a standardized unit defined under strict conditions to allow for accurate comparisons between species. This includes that BMR should be measured in an organism's thermoneutral zone (TNZ), but a TNZ has not been determined for many aquatic species (Rosen & Trites, 2014) and it is therefore unclear which studies provide “true” estimates of BMR. In one study that adhered strictly to all conditions for BMR other than the TNZ, the authors found no evidence of differences in BMR between aquatic and terrestrial mammals (Lavigne et al., 1986).

Second, heteroscedasticity, or the nonconstant variance of regression residuals, could influence analyses of the scaling of BMR. Heteroscedasticity can be seen in the “mouse to elephant” scatter plot/regression in studies investigating metabolic scaling, with BMR generally being underestimated at higher *M*_b_ (Kolokotrones et al., 2010; McNab, 1988). Given that all fully aquatic mammals weigh more than 10 kg, their BMR may appear elevated relative to the best fit regression line, falsely giving the impression that they have elevated metabolisms for their size. A previous study attempted to account for this heteroscedasticity by using a quadratic equation to estimate the allometric relationship in terrestrial mammals (Kolokotrones et al., 2010). However, the quadratic model's accuracy is not consistent across the entire body mass range, as it becomes isometric for *M*_b_ above approximately 700 kg, which results in the prediction that the BMR of a blue whale (190 tonne) would be 388% higher than that estimated from the equation by Kleiber (1961).

Third, studies of allometric scaling of BMR in terrestrial versus aquatic species can only be understood within the limitations of the data used and/or analyses employed (Hennemann, 1983), which can either generate false differences or mask true differences if they are used improperly. In the case of aquatic mammals, many studies may have been measuring resting metabolic rate (RMR), where conditions are more relaxed while the animal is at rest, rather than BMR, and thus reporting overestimates of the latter (Kasting et al., 1989). Last, no previous studies comparing the allometric scaling of BMR in aquatic and terrestrial mammals have accounted for phylogeny, thus violating a key assumption of regression analyses that the data points are independent.

Aerobic metabolism requires adequate oxygen delivery to the cells. In this study we address how the O_2_ necessary for basal metabolism is supplied by the cardiorespiratory system by providing the first allometric equations relating metabolism to cardiorespiratory variables. Blood pumped by the heart—cardiac output—provides O_2_ to the body, and thus we hypothesize that cardiac output should be modulated with basal energetic requirements during periods when an animal's metabolic rate is basal. Likewise, minute ventilation, which measures how much air is exchanged in the lungs per minute, should also be regulated in accordance with metabolic demands (Bruce, 2017). To test these hypotheses, we gathered data from the literature to compare the allometry of cardiorespiratory flow of O_2_ with BMR in aquatic versus terrestrial mammals.

**FIGURE 1:**
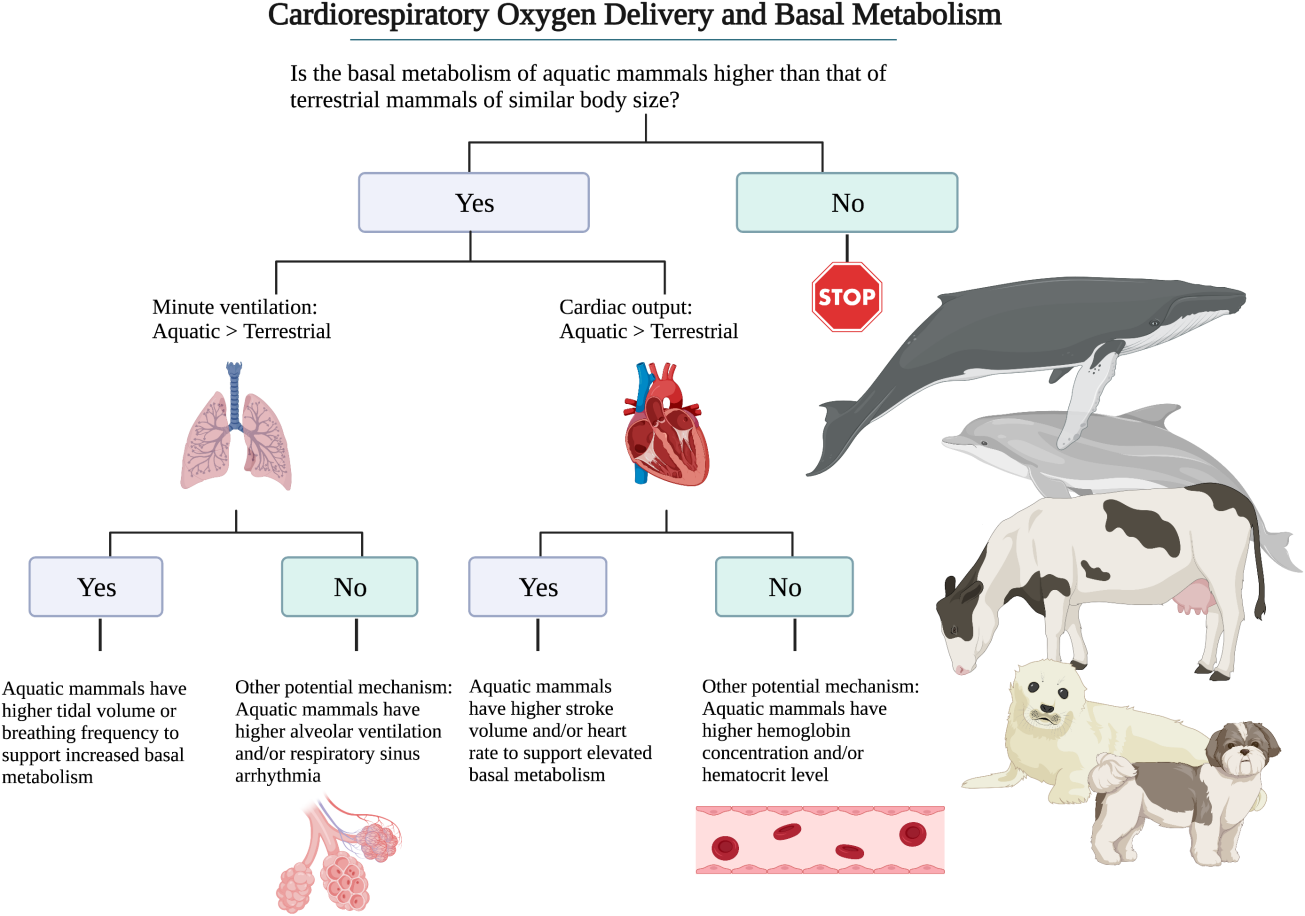
The hypotheses tested in the current study. Oxygen flow from the atmosphere to the cell is hypothesized to correlate to any observed differences of basal metabolic rate (BMR) scaling between aquatic versus terrestrial mammals. Created with BioRender.com

For our analyses, we use a dataset of BMR assembled for aquatic and terrestrial mammals in combination with phylogeny. In addition to calculating and comparing scaling relationships of BMR and *M*_b_, we test the hypothesis that if BMR differs between aquatic and terrestrial species, so should the cardiorespiratory delivery of O_2_ via differences in the scaling of minute ventilation and cardiac output. To achieve this, we use empirical data for tidal volume (*V*_T_), breathing frequency (*f*_R_), heart rate (*f*_H_), and stroke volume (SV) to estimate whether the allometry of minute ventilation (*V*_T_·*f*_R_) and cardiac output (*f*_H_·SV) agree with the allometry of aquatic and terrestrial BMR. Specifically, we test whether (1) BMR is higher in aquatic mammals than terrestrial mammals, and, if so, whether this higher BMR is supported by (2) an elevated delivery of O_2_ through the cardiorespiratory system during periods of rest (Figure 1).

## 2 MATERIALS AND METHODS

As information about the conditions under which metabolic and physiological measurements were made is not always available or addressed in primary sources, we refer to these measurements as the best estimated BMR given the available information in the literature. We only included measurements that had been obtained at a (water or air) temperature range which is reported for the species in the wild. For cardiorespiratory variables, no standardized criteria exist for their measurement, so we elected to only include data for inactive, awake (not sedated), adults.

### 2.1 Basal metabolic rate

Basal metabolic rates from 43 terrestrial and 20 aquatic mammal species were compiled (Table 1) from previously published datasets (Genoud et al., 2018; McNab, 2008; White & Seymour, 2003; White & Seymour, 2005). All data were obtained from species with a *M*_b_ of ≥10 kg, representing the *M*_b_ of the smallest aquatic mammal, and ≤5318 kg for BMR and ≤6650 kg for cardiovascular variables. We therefore excluded many smaller terrestrial mammals that have been included in previous studies to avoid an unequal distribution of aquatic and terrestrial mammals. The criteria for BMR were (1) inactive (2) adults in a (3) postabsorptive, and (4) nonreproductive state, measured under (5) thermoneutral conditions (Kleiber, 1961). Most data from aquatic mammals were measured in water, although some measurements of pinnipeds were made on land. For a few species, not all criteria for BMR could be verified; the reported measurement conditions are detailed in the supplementary material.

**TABLE 1:** The total (terrestrial + aquatic) number of orders, families, genera, and species represented in terms of basal metabolic rate (BMR), breathing frequency (*f*_R_), tidal volume (*V*_T_), heart rate (*f*_H_), and stroke volume (SV). Number in parenthesis is the number for aquatic mammals. Average body mass (*M*_b_, kg) is stated, with the range in parentheses. The data for this table can be found in the supplementary materials.

Full data set	Order	Family	Genus	Species	Mean *M*_b_ (range)	
					Terrestrial	Aquatic
BMR	13 (4)	32 (10)	55 (18)	63 (20)	154 ± 585 (10–3833)	483 ± 1187 (10–5318)
*f*_R_	7 (4)	25 (13)	62 (24)	76 (26)	464 ± 875 (12–4550)	819 ± 1466 (11–6650)
*V*_T_	6 (3)	14 (6)	23 (12)	26 (13)	183 ± 184 (16–550)	856 ± 1423 (81–4905)
*f*_H_	7 (4)	19 (11)	25 (17)	42 (20)	722 ± 1299 (18–5000)	601 ± 698 (11–2621)
SV	5 (2)	11 (3)	15 (4)	15 (4)	817±1376 (25–4080)	1310 ± 2137 (36–4500)

### 2.2 Cardiorespiratory variables

We collected previously published data for *f*_H_, SV, *V*_T_, and *f*_R_ measured on inactive, awake, adult aquatic, and terrestrial mammals (Table 1). The number of species included in each group, and the average *M*_b_ of each of those species are summarized in Table 1. We regressed each of these cardiorespiratory variables against *M*_b_ for mammals from 11 to 6650 kg.

### 2.3 Statistical analysis

Brooks the allometric relationships between dependent variables (BMR, *f*_H_, *f*_R_, *V*_T_, and SV) and the independent variables, *M*_b_, and habitat (aquatic or terrestrial) were analyzed using a general linear mixed‐effects (GLM) model with nested random effects of order and (where possible) family and genus to account for phylogeny. The mixed‐effect model approach is an alternative to, and in a sense a generalization of, the phylogenetic generalized least squared (PGLS) approach; it also accounts for phylogeny, but additionally allows estimation of residual variance and inter‐taxon variances separately and generalizes smoothly beyond the normal, linear, univariate case. This approach has been reviewed by de Villemereuil and Nakagawa (2014) with applications  in a number of studies (Adams & Nason, 2018; Castillo, 2017; Kumar et al., 2021; Martin et al., 2020; Volf et al., 2018). Models were fitted in R statistical computing software (R Core Team, 2021; RStudio Team, 2021) using the *glmmTMB* package; all code and data files are included in the supplementary materials. For all analyses, BMR (kcal·day^−1^), *f*_H_ (beats·min^−1^), *f*_R_ (breaths·min^−1^), *V*_T_ (mL), and *M*_b_ (kg) were transformed using the base 10 logarithm (log_10_) (Glazier, 2021). Models included an interaction term between habitat and *M*_b_ to determine whether there were differences in the slopes for aquatic and terrestrial mammals. For each model, after confirming that model assumptions were met for linearity, residual normality, independence, and constant variance, a Type II ANOVA was performed to assess the contributions of each predictor (supplementary materials).

### 2.4 Estimating cardiac output and minute ventilation

We used the regression equations for *V*_T_, *f*_R_, *f*_H,_ and SV, and a parametric bootstrap, to obtain model predictions with uncertainty estimates for allometric equations of cardiac output (CO = SV·*f*_H_) and respiratory minute ventilation (MV = *V*_T_·*f*_R_) for aquatic and terrestrial mammals separately. Described in brief: we used the function *predict.glmmTMB* from the *glmmTMB* R package (Brooks et al., 2017), and function *bootMer* from the *lme4* R package (Bates et al., 2015) to compute 100 parametric bootstrap predictions of *V*_T_, *f*_R_, *f*_H,_ and SV for each species in the dataset, incorporating uncertainty in model parameter estimates and random effects of order and (where estimated) family and genus. The parametric bootstrap samples were multiplied to estimate CO and MV (e.g. CO = SV·*f*_H_), and the results were used to obtain predicted values with percentile‐based 95% confidence intervals (see section 6 in the supplementary material for code details). We followed a similar procedure to obtain estimates and confidence intervals for the expected difference of the allometric equations in CO and MV for hypothetical species of varying masses differing only in habitat (aquatic vs. terrestrial). Population‐level predictions were used for these differences (i.e., neglecting all random effects; see sections 7.1 and 7.2 in supplementary material for code details).

## 3 RESULTS

### 3.1 Basal metabolic rate

**FIGURE 2:**
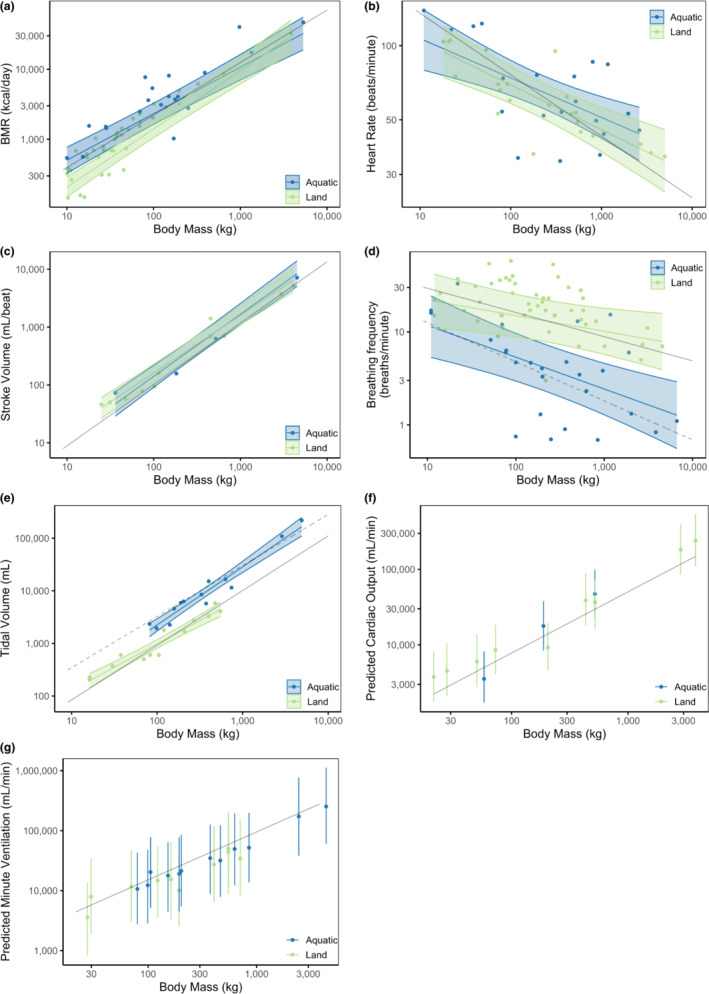
(a) Basal metabolic rate (BMR, kcal·day^−1^), (b) heart rate (beats·min^−1^), (c) stroke volume (mL·beat^−1^), (d) breathing frequency (breaths·min^−1^), (e) tidal volume (mL·breath^−1^), (f) cardiac output (mL·min^−1^), (g) minute ventilation (mL·min^−1^), against body mass (kg) for aquatic and terrestrial mammals. The solid (terrestrial) and broken (aquatic) black lines are prediction equations from the literature: (a) BMR terrestrial = 70·body mass^0.75^ (Kleiber, 1947), (b) heart rate terrestrial = 241·body mass^−0.25^ (Stahl, 1967), (c) stroke volume terrestrial = cardiac output/heart rate = [181·body mass^0.81^]/[241·body mass^−0.25^]^−1^ (Stahl, 1967), (d) breathing frequency terrestrial = 53.5·body mass^−0.26^ (Stahl, 1967), breathing frequency aquatic = 33·body mass^−0.42^ (Mortola & Limoges, 2006), (e) tidal volume terrestrial = 7.69·body mass^1.04^ (Stahl, 1967), tidal volume aquatic = 0.0372·body mass^0.92^ (Fahlman et al., 2020), (f) cardiac output = 187·body mass^0.81^ (Stahl, 1967), and (g) minute ventilation = 379·body mass^0.80^ (Stahl, 1967). In panels a‐d, dots are observed data points and colored lines with shaded regions are population‐average model predictions with 95% confidence intervals. In panels e‐f, colored dots with error bars are mean values and percentile‐based 95% confidence intervals from parametric boostrap.

Our analyses indicate that the BMR of small aquatic mammals (*M*_b_ < 100 kg) is higher than for terrestrial mammals of the same size, but the BMR is the same for species with *M*_b_ ≥ 100 kg, regardless of their habitat (Figure 2a). There were significant differences in both the intercepts (terrestrial = 1.49; aquatic = 2.04) and slopes (terrestrial = 0.83; aquatic = 0.66) for the allometric equations of BMR for aquatic and terrestrial mammals (Table 2, Figure 2a, see Appendix section 1.2). For example, the model indicated that BMR was 140% higher in aquatic mammals than their terrestrial counterparts at 10 kg but 17% lower at 5000 kg, suggesting differences in the scaling of BMR in aquatic versus terrestrial mammals.

**TABLE 2:** Allometric model results from general linear mixed‐effects model (GLMM) model with nested random effects for order and, where possible, family and genus, to estimate log_10_‐transformed (log_10_) basal metabolic rate (BMR, kcal·day^−1^), heart rate (*f*_H_, beats·min^−1^), stroke volume (SV, mL), breathing frequency (*f*_R_, breaths·min^−1^), and tidal volume (*V*_T_, mL). Explanatory variables include log_10_‐transformed body mass ([log_10_]*M*_b_), habitat (a parameter that alters the intercept for terrestrial mammals), and a cross‐term for log_10_‐transformed *M*_b_ and habitat (the slope). Table includes parameter estimates (±SE, with confidence limits in parenthesis) of the full model.

Model	Intercept	[log_10_]*M*_b_	Habitat (terrestrial)	[log_10_]*M*_b_ × habitat	Appendix section
Reduced model					
[log_10_]BMR	2.04 ± 0.14 (1.77:2.30)	0.66 ± 0.05 (0.56:0.77)^†^	−0.55 ± 0.13 (−0.81:‐0.29)^†^	0.17 ± 0.07 (0.03:0.31)*	1.2
[log_10_]*f*_R_	1.41 ± 0.24 (0.95:1.88)	−0.34 ± 0.08 (−0.51:‐0.17)^†^	0.10 ± 0.26 (−0.41:0.62)^†^	0.17 ± 0.11 (−0.04:0.39)	2.2
[log_10_]*f*_H_	2.19 ± 0.10 (1.99:2.39)	−0.16 ± 0.04 (−0.24:‐0.08)^†^	0.01 ± 0.14 (−0.26:0.29)	−0.02 ± 0.06 (−0.13–0.09)	3.2
[log_10_]SV	−0.002 ± 0.0.19 (−0.36:0.37)	1.07 ± 0.06 (0.95:1.20)^†^	0.20 ± 0.20 (−0.18:0.59)	−0.07 ± 0.08 (−0.23:0.08)	4.2
[log_10_]*V*_T_	1.14 ± 0.18 (0.78:1.51)	1.10 ± 0.07 (0.98:1.24)^†^	0.07 ± 0.24 (−0.40:0.55)	−0.22 ± 0.10 (−0.42:−0.02)*	5.2
Full model					
[log_10_]BMR	2.04 ± 0.14 (1.77:2.30)	0.66 ± 0.05 (0.56:0.77)^†^	−0.55 ± 0.13 (−0.81:−0.29)^†^	0.17 ± 0.07 (0.03:0.31)*	1.2
[log_10_]*f*_R_	1.24 ± 0.20 (0.84:1.64)	−0.28 ± 0.07 (−0.42:‐0.14)^†^	0.24 ± 0.24 (−0.22:0.71)^†^	0.13 ± 0.10 (−0.07:0.32)	2.2
[log_10_]*f*_H_	2.28 ± 0.10 (2.07:2.49)	−0.19 ± 0.04 (−0.27:‐0.11)^†^	−0.02 ± 0.16 (−0.33:0.29)	0.02 ± 0.06 (−0.11–0.14)	3.2
[log_10_]SV	−0.08 ± 0.21 (−0.48:0.32)	1.08 ± 0.07 (0.93:1.23)^†^	0.20 ± 0.24 (−0.26:0.66)	−0.06 ± 0.10 (−0.25:0.13)	4.2
[log_10_]*V*_T_	1.51 ± 0.18 (1.15:1.87)	0.97 ± 0.07 (0.83:1.10)^†^	−0.19 ± 0.23 (−0.65:0.27)	−0.13 ± 0.10 (−0.32:0.07)	5.2

*Note*: * and † indicates significant *p*‐value either <0.05 or <0.01, respectively, of each specific parameter (using a Type II Wald chi‐square test). Additional results can be found in the Appendix. The reduced model is the one considered for this study and excludes juvenile mammals and those sedated during the study. The full model includes juvenile and sedated mammals and is used for comparison only in this table.

### 3.2 Heart rate and stroke volume

There was no significant difference between aquatic and terrestrial mammals in the scaling of *f*_H_ (Table 2, Figure 2b, see Appendix section 3.2). Similarly, there were no significant differences in intercepts or slopes for allometric equations of SV between aquatic and terrestrial mammals (Table 2, Figure 2c, see Appendix section 4.2). Together, these models offer little evidence of an association between habitat and *f*_H_ or SV after accounting for *M*_b_.

### 3.3 Breathing frequency and tidal volume

For terrestrial mammals, the intercept for the allometric equation for *f*_R_ was higher than for aquatic mammals (terrestrial: 1.51; aquatic: 1.41) while the slope was similar (Table 2, Figure 2d). At 10 and 5000 kg, *f*_R_ was 61 and 81% lower, respectively, in aquatic than terrestrial mammals (Table 2, Figure 2d). On the other hand, the slope of the allometric equation for *V*_T_ was lower in terrestrial mammals (terrestrial: 0.89; aquatic: 1.10) (Table 2, Figure 2d and e), and *V*_T_ was 90 and 602% higher in a 10 and 5000 kg aquatic mammal, respectively, than in terrestrial mammals of the same size (Table 2, Figure 2e). These analyses support the notion that *f*_R_ and *V*_T_ are associated with both *M*_b_ and habitat, such that aquatic mammals demonstrate elevated *f*_R_ and *V*_T_ compared to similar‐sized terrestrial mammals.

### 3.4 Estimating cardiac output and minute ventilation

Parametric bootstrapping (see section 6) was used to estimate CO (Figure 2f) and MV (Figure 2g) based on the random‐effects model (Table 2). The differences in the resulting CO and MV allometric equations between aquatic and terrestrial mammals were computed using a parametric bootstrap (see Appendix section 7). For both the estimated CO (Appendix Figures 7.2 and 7.4) and MV (Appendix Figures 7.6 and 7.8), the confidence limits for aquatic and terrestrial species overlapped strongly, with the confidence interval for the difference spanning zero, indicating that for aquatic and terrestrial groups listed in our dataset there is not good evidence for an association between CO or MV and habitat.

## 4 DISCUSSION

Our findings show that aquatic and terrestrial mammals >10 kg differ in both the slope and intercept of BMR and *M*_b_, though only in smaller species (*M*_b_ < 100 kg). Our decision to limit the dataset to mammals ≥10 kg may provide an explanation for previous contradictory results for scaling of BMRs in aquatic versus terrestrial mammals and underscores the importance of analysis with a balanced dataset (Genoud et al., 2018; McNab, 1988). We hypothesized that differences in BMR between aquatic and terrestrial mammals were associated with concomitant changes in the delivery of O_2_ via the cardiorespiratory system (i.e., increased CO and MV in aquatic species given that their BMRs are elevated). However, our analysis suggested that neither the allometry of CO nor MV differs between aquatic and terrestrial mammals, despite small aquatic mammals having higher BMRs than equivalently‐sized terrestrial mammals.

### 4.1 Higher BMRs in small aquatic mammals

Several hypotheses have been proposed to explain the observed allometric relationship between BMR and *M*_b_. One hypothesis is based on the geometric principles that body volume and surface area change disproportionally with increasing size. This results in an elevated surface‐area to volume ratio in smaller animals, which may lead to a disproportionately higher heat loss (Rubner, 1865; White & Seymour, 2003). Thus, thermoregulation would be particularly challenging for small animals in water, a medium with 25 times higher heat capacity than air. Although many aquatic species have anatomical adaptations to reduce heat loss, such as large adipose stores and countercurrent heat exchangers, these adaptations alone may not be sufficient for small aquatic mammals to remain thermally stable. They may therefore have to compensate for the higher heat loss in water through production of metabolic heat. One potential mechanism by which increased basal metabolic activity could result from increased thermoregulatory capacity is via the mitochondrial proton leak in the locomotory muscles of smaller aquatic mammals (Wright et al., 2021), whereby heat is produced rather than ATP from oxidative phosphorylation in the mitochondria.

### 4.2 How is elevated BMR supported in aquatic mammals?

Our analyses suggest that CO and MV are unable to fully explain increased O_2_ delivery to the heart to support higher BMR in small aquatic mammals. Thus, other mechanisms may exist that provide sufficient O_2_ to support an elevated BMR. The lower *f*_R_ of aquatic mammals has been proposed to help regulate buoyancy rather than having a respiratory function (Mortola, 2015). In general, *f*_R_ relates negatively to *M*_b_. However, our results show *f*_R_ decreases faster with *M*_b_ in aquatic mammals than terrestrial animals (Figure 2d), revealing the first component of the aquatic (apneustic) breathing strategy of aquatic species, characterized by brief breaths followed by prolonged inter‐breath intervals (Fahlman et al., 2017). The second component of the strategy is that *V*_T_, in contrast, is elevated in aquatic species (Figure 2e), but this is due to differing slopes (aquatic: 1.10, terrestrial: 0.89) rather than intercepts (Table 2). That is, smaller aquatic and terrestrial mammals take similar‐sized breaths, whereas large aquatic mammals take larger breaths than large terrestrial mammals. Comprehensively, the aquatic breathing strategy, with a lower *f*_R_ and higher *V*_T_, has been proposed to improve gas exchange by inducing a higher alveolar ventilation (${\dot{V}}_{\;A}$). As ${\dot{V}}_{\;A}$ accounts for dead space ventilation, it more accurately reflects the air that reaches the alveoli and is used for gas exchange, whereas MV reflects the amount of air moving in and out of the respiratory system. As aquatic mammals tend to have a larger dead space (7% in aquatic mammals as compared with 3% in terrestrial mammals, Kooyman, 1973, Stahl, 1967), they have a greater dead space ventilation. Taking fewer and deeper breaths may be one strategy to reduce this dead space ventilation, which will increase ${\dot{V}}_{\;A}$ and thereby improve gas exchange without necessitating an increase in MV (Fahlman et al., 2017).

Additionally, marine mammals tend to have higher blood O_2_ loading capacity due to higher hematocrit and hemoglobin concentrations (Castellini et al., 2010). This is thought to be an adaptation to increase their blood O_2_ stores for diving, lengthening aerobic dive durations (Beechler et al., 2009; Choy et al., 2019; Vestweber et al., 1991). Thus, the same volume of blood ejected from the heart should contain more O_2_ in an aquatic mammal. This difference would be reflected in the cardiac O_2_ output ($\dot{C}O_{O_{2}}$, L O_2_·min^−1^) which can be calculated as:

(1) $$\dot{C}O_{O_{2}}\left(L\;O_{2}\cdot \min^{-1}\right)=f_{H}\cdot \mathrm{SV}\cdot \mathrm{Hb}\cdot 1.34\cdot 0.01$$

where Hb is the hemoglobin concentration (g·dL^−1^) and 1.34 is the O_2_ loading capacity of hemoglobin (mL O_2_ per g hemoglobin). The $\dot{C}O_{O_{2}}$ is a more appropriate measure of the capacity of the cardiovascular system to deliver O_2_, and we suggest it should be used in future studies of allometric comparison. As an example, the published hemoglobin concentration for a beluga (23 g·dL^−1^, Choy et al., 2019), horse (15.5 g hemoglobin·dL^−1^, Plotka et al., 1988), and cow (9.6 g·dL^−1^, Rusoff et al., 1951) suggests that the same volume of blood delivers 82% and 33% more O_2_ in the beluga in comparison to similar‐sized cows and horses, respectively. Taken together, our analyses suggest that the aquatic breathing strategy and higher blood O_2_ loading capacity of aquatic mammals could provide the means for increased O_2_ delivery in small aquatic mammals for their elevated basal metabolism. For large aquatic mammals, these adaptations could enable a greater aerobic scope.

### 4.3 Broader applications to metabolism, conservation, and hypoxia‐related diseases

The implications of our study are threefold: these results can (1) be applied to understand metabolism across multiple levels of biological organization, (2) provide an improved understanding of basal energetics for aquatic mammals, and (3) can be used to make predictions for larger, difficult‐to‐study aquatic, and terrestrial species. Because BMR is a standardized measurement, it may be used in predicting field metabolic rate (FMR), which is typically two to three times higher than BMR (Allen, 2021). In turn, improved estimates of daily energy requirements can provide the basis to estimate metabolic needs at a population level which reflect energy/bioenergetic flow in populations. Given that many species of aquatic mammals are threatened, vulnerable, or close to extinction and only limited knowledge of their energetic needs are known, metabolic information gained from allometric scaling can help provide baseline information that is necessary to understand the impacts of climate change and human‐induced disturbance on aquatic species.

In addition, exploring the physiological adaptations of species evolved to thrive in challenging environments can inspire novel approaches to treating diseases that are secondary to conditions that mimic environmental stressors. We have suggested that ${\dot{V}}_{\;A}$, secondary to the unique breathing strategy of aquatic mammals, helps to support an enhanced aerobic capacity. This may be important to support recovery from acute hypoxia and declining partial pressures, which are known to harm oxygen‐sensitive human tissues during repeated dives by aquatic mammals (Williams & Davis, 2021). Aquatic mammals' adaptations for defense against hypoxia have been discussed in the context of hypoxia‐related symptoms associated with COVID‐19 and other respiratory illnesses—for example, adaptations of increased blood volume, hemoglobin, hematocrit, and oxygen carrying capacity may address hypoxia‐related symptoms in the vascular system like thrombosis and coagulopathy, whereas increased myoglobin concentration and glycogen tissue store may address hypoxia‐related symptoms in the cardiac and skeletal muscles such as cardiac arrhythmias, muscle fatigue, and sinus tachycardia (Williams & Davis, 2021). Examining the functional interactions of physiological variables of aquatic mammals allows us to surmise about ways to mimic these adaptations in terrestrial mammals to better adapt to extreme conditions. Importantly, this highlights the utility of understanding how the cardiovascular and respiratory systems support metabolic requirements for comparative medicine.

In summary, our analysis shows that aquatic mammals exhibit an elevated BMR compared to terrestrial mammals at smaller, but not larger, *M*_b_'s. An elevated BMR requires increased O_2_ delivery to the cells, which we argue is supported by the aquatic breathing strategy, where aquatic mammals exhibit higher *V*_T_ and lower *f*_R_, coupled with higher blood O_2_ capacity. This improves ${\dot{V}}_{\;A}$ and gas exchange, and increases $\dot{C}O_{O_{2}}$ . These findings demonstrate how comparative physiology can provide valuable insights elucidating how metabolic homeostasis is maintained in different environments.

## AUTHOR CONTRIBUTIONS

AF and JAS conceived the study. JAS, AB, and AF developed the hypothesis. RSH and DLD obtained the historical data. RSH, SDR, TW, DLD, AS, BS, SD, and AF analyzed the data. SDR, TW, AS, BS, and SD carried out the statistical analysis. RSH and AF drafted the paper with feedback from co‐authors. All authors gave final approval for publication.

## FUNDING INFORMATION

JAS and AF acknowledge funding support from the Office of Naval Research.

## Supporting information


Appendix S1.
