# Supplementary material for: Allometric scaling of metabolic rate and cardiorespiratory variables in aquatic and terrestrial mammals
Source: Physiol Rep. 2023 Jun 4;11(11):e15698. doi: 10.14814/phy2.15698 (PMC10239733; doi:10.14814/phy2.15698)
Supplement: Supplementary file 1 — Appendix S1. [file PHY2-11-e15698-s001.docx]

Appendix: [https://stacyderuiter.github.io/mammal-allometry/supplement.html](https://urldefense.com/v3/__https:/stacyderuiter.github.io/mammal-allometry/supplement.html__;!!N11eV2iwtfs!pp8GzAU7JZ4TQZNwW8oy54xf2eRUdHeRB79MK1zh-SOnuggzme4v8V_vY6YNYGnJ1pId2QMH2oY$)

Supplementary material: [https://stacyderuiter.github.io/mammal-allometry/](https://urldefense.com/v3/__https:/stacyderuiter.github.io/mammal-allometry/__;!!N11eV2iwtfs!pp8GzAU7JZ4TQZNwW8oy54xf2eRUdHeRB79MK1zh-SOnuggzme4v8V_vY6YNYGnJ1pIdIuNfIx4$)
